# Is there an association between skeletal asymmetry and tooth absence?

**DOI:** 10.1590/2177-6709.21.4.073-079.oar

**Published:** 2016

**Authors:** Guilherme Thiesen, Bruno Frazão Gribel, Keila Cristina Rausch Pereira, Maria Perpetua Mota Freitas

**Affiliations:** 1 Professor of Orthodontics, Universidade do Sul de Santa Catarina (UNISUL), Florianópolis, Santa Catarina, Brazil.; 2 MSc, Pontifícia Universidade Católica de Minas Gerais (PUC-MG), Belo Horizonte, MG, Brazil.; 3 Professor of Epidemiology, Universidade do Sul de Santa Catarina (UNISUL), Florianópolis, SC, Brazil.; 4 Professor of Orthodontics, Universidade Luterana do Brasil (ULBRA), Canoas, RS, Brazil.

**Keywords:** Facial asymmetry, Tooth loss, Epidemiology, Orthodontics

## Abstract

**Introduction:**

Facial skeletal asymmetry is commonly found in humans and its main characteristic is menton deviation. The literature suggests that occlusal and masticatory problems arising from tooth absence could be related to the development of such asymmetries.

**Objective:**

The aim of this cross-sectional study was to estimate the prevalence of mandibular skeletal asymmetries and to investigate its association with posterior tooth absences.

**Methods:**

Tomographic images of 952 individuals aged from 18 to 75 years old were used. Asymmetry was the analyzed outcome, and it was categorized into three groups according to gnathion displacement in relation to the midsagittal plane (relative symmetry, moderate asymmetry, and severe asymmetry). Patients were sorted by the presence of all posterior teeth, unilateral posterior tooth absence, or bilateral posterior tooth absence. Chi-square test with a significance level of 5% was used to verify the association between posterior tooth absence and asymmetry.

**Results:**

Results show relative symmetry present in 55.3% of the sample, as well as the prevalence of 27.3% for moderate mandibular asymmetry and 17.4% for severe asymmetry. Moderate and severe mandibular asymmetries occurred in a higher proportion in patients with unilateral posterior tooth absence. However, there was no statistically significant difference between the analyzed groups (*p* = 0.691).

**Conclusions:**

In this study, mandibular asymmetries did not present any association with the absence of teeth on the posterior area of the arch.

## RESUMO

**Introdução:**

a assimetria esquelética facial é comum em humanos, sendo o desvio do mento sua principal característica. É sugerido, na literatura, que problemas oclusais e mastigatórios advindos das ausências dentárias teriam relação com o desenvolvimento dessas assimetrias.

**Objetivo:**

o objetivo deste estudo transversal foi estimar a prevalência de assimetrias esqueléticas mandibulares e investigar sua associação com as ausências dentárias posteriores.

**Métodos:**

foram utilizadas imagens tomográficas de 952 indivíduos, com idade entre 18 e 75 anos. A assimetria foi o desfecho analisado, sendo categorizada em três grupos, de acordo com o desvio do gnátio em relação ao plano sagital mediano: simetria relativa, assimetria moderada e assimetria severa. Os indivíduos foram agrupados segundo a presença de todos os dentes posteriores, ausência dentária posterior unilateral ou ausência dentária posterior bilateral. Para verificar a associação entre a ausência dentária posterior e a assimetria, foi utilizado o teste Χ^2^, ao nível de significância de 5%.

**Resultados:**

os resultados mostraram que a simetria relativa esteve presente em 55,3% da amostra, bem como uma prevalência de 27,3% para a assimetria mandibular moderada e 17,4% para assimetria severa. As assimetrias mandibulares moderada e severa ocorreram em maior proporção nos indivíduos com ausência dentária posterior unilateral; entretanto, não houve diferença estatisticamente significativa entre os grupos (*p* = 0,691).

**Conclusões:**

nesse estudo, as assimetrias mandibulares em adultos não apresentaram associação com a ausência de dentes na região posterior da arcada dentária.

## INTRODUCTION

The human body tends to present symmetric skeletal development, which implies that both right and left sides should have the same size and shape. However, asymmetry is commonly found in the general population. This is observed by bilateral disharmonies in the craniofacial complex that may not be associated with syndromes, traumas, or pathologies.^1^ ^,^ ^2^ In mild degrees, such skeletal asymmetries may go unnoticed. Nevertheless, moderate and severe degrees may require orthodontic or orthopedic correction, or even orthognathic surgery.^3^

Severt and Proffit,^4^ using a sample of 1460 patients assessed at North Carolina University, reported that 34% of patients presented facial asymmetry. Menton deviation was found in 74% of patients considered asymmetric, thus being the strongest characteristic of asymmetry. 

Several studies investigated skeletal asymmetries in patients without missing teeth by means of different methods.^1^ ^,^ ^5^ ^-^ ^9^ Nevertheless, the effects of posterior tooth absence over skeletal asymmetries are conflicting, and only a few studies have approached this subject; only a few epidemiological studies with significant samplings can be found. Some authors^10^ ^-^ ^15^ argue that absence of posterior teeth may cause malocclusion, tipping of adjacent teeth towards the extraction area, extrusion of antagonist teeth, or unilateral mastication habits. Such occlusal and functional problems deriving from tooth absence could be associated with the development of skeletal asymmetries.

Therefore, the aim of this cross-sectional study was to estimate the prevalence of mandibular skeletal asymmetries in adults and evaluate their association with posterior tooth absence. The study was based on the hypothesis that decreased masticatory function caused by tooth absence could be related to such asymmetries.

## MATERIAL AND METHODS

The Ethics Committee of Universidade Luterana do Brasil (ULBRA, Canoas, RS, Brazil) approved this study under protocol number #771293 on August 28^th^, 2014. 

The sample was composed of cone-beam computed tomography (CBCT) of 952 individuals, pertaining to the database of a service center for dental diagnosis and planning (Compass3D, Belo Horizonte, MG, Brazil), which receives tomographic images from all over the country. Such images were obtained between the years of 2012 and 2013, and there was a random choice in regards to sex and race of the sample. This random selection regarding race was chosen due to the current difficulty to racially segregate the Brazilian population, given its mixed ethnic ancestry and its multidimensional categorizing. 

For sample size calculation, a pilot study with 100 randomly chosen individuals was conducted in order to obtain the proportion of mandibular asymmetry (moderate and severe) on people with tooth presence or tooth absence. In order to do so, Epi Info version 7 software (CDC, Atlanta, GA, USA) was used, evaluating the association between the exposition factor and mandibular asymmetry. There was an expected prevalence of 44% of alterations (mandibular asymmetry) in unexposed patients, using a 95% confidence interval and 80% statistical power. Assuming a proportion of unexposed (all teeth present) to exposed (tooth absences) of 2.2:1 and a minimal prevalence ratio of 1.3, the minimal sample required would be of 784 patients, according to the Fleiss method with correction for continuity.^16^

The following inclusion criteria were adopted (based on the medical records requiring tomographic examination): tomographic scans requested under proper clinical justification, or due to the impossibility of meeting clinical necessities by means of conventional radiographic techniques, thus following the guidelines of the SedentexCT project and the American Academy of Oral and Maxillofacial Radiology;^17^ ^,^ ^18^ patients aged from 18 to 75 years old; and images obtained by the same brand of tomographic device (i-CAT, Imaging Sciences International, Hatfield, PA, USA). Exclusion criteria were determined by the medical records and analysis of panoramic reconstructions, as follows: prior history of facial fractures and/or surgery; degenerative disease on the temporomandibular joint; craniofacial syndromes and anomalies; completely edentulous patients; and patients subjected to orthodontic treatment with four extracted teeth, dental implants, or the use of attached partial prosthesis.

All tomographic scans were obtained by i-CAT device, adjusted to operate under the following specifications: extended field of view (FOV 16 cm x 22 cm or 17 cm x 23 cm), 120 KvP, 3-8 mA and typical 0.4 mm voxel size. Patients were asked to occlude at maximal intercuspation and relax the lips; they were also advised to sit and position the head according to the Frankfurt plane (parallel to the floor) and the midsagittal plane (perpendicular to the floor). 

CBCT scans were exported in DICOM (Digital Imaging and Communication in Medicine) format, using i-CAT Vision software. The DICOM files were loaded into SimPlant Ortho Pro 2.0 software (Materialise Dental, Leuven, FB, Belgium) which is capable of providing exact values for the measurements of choice. In order to improve measurement precision, anatomical landmarks were located by means of multiplanar reconstruction slices and a measurement scale of 0.01 mm. 

Patients were characterized as to tooth presence by means of panoramic reconstruction carried out with SimPlant Ortho Pro 2.0 software by the same calibrated examiner at all times. Patients presenting all erupted permanent teeth from first premolar to second molar, on all four quadrants, were classified as having all teeth. Those with at least one unilateral missing tooth from first premolar to second molar, regardless if it were located in the upper, lower or both jaws, were considered as having unilateral tooth absence. If absence occurred on both right and left sides of patient's dentition, tooth absence was considered bilateral. Residual roots were treated as tooth absence. Single fixed root-supported prostheses were considered as tooth presence. 

**Figure 1:**
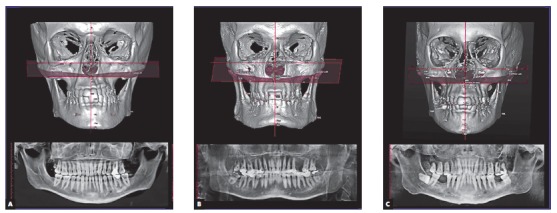
Patients affected by different degrees of mandibular asymmetry that presented all posterior permanent teeth (A), unilateral posterior tooth absence (B), and bilateral posterior tooth absence (C).

The outcomes were categorized into three groups according to the degree of mandibular asymmetry defined by analysis of menton deviation, since this factor exerts the biggest influence over the perception of facial symmetry.^4^ ^,^ ^8^ ^,^ ^19^ It was determined by gnathion displacement in relation to patient's midsagittal plane, regardless of the side of deviation. Patients with gnathion displacement of up to 2 mm were considered as having a relative symmetry.^5^ ^,^ ^8^ ^,^ ^9^ ^,^ ^20^ Patients with displacement greater than 2 mm and less than 4 mm were classified as having moderate asymmetry. Finally, patients with gnathion displacement greater than 4 mm in relation to the midsagittal plane were classified as having severe asymmetry^6^ ^,^ ^8^ ^,^ ^19^ (Fig 1).

Anatomical landmarks were determined (Table 1) to establish the following reference planes: 

**Table 1:** Landmarks selected for the study.

Landmark	Anatomical region	Sagittal view	Axial view	Coronal view
Porion (Po)	External auditory meatus of the ear canal	Middle-superior-most point on the external auditory meatus	Middle-superior-most point	Superior-most point
Orbitale (Or)	Latero-inferior contour of the orbit	Anterior-superior-most point on the edge between the internal and external contours	Anterior-most point	Latero-inferior most point
Basion (Ba)	Anterior margin of the foramen magnum	Inferior-most point	Anterior-most point	Middle-anterior-most point
Nasion (N)	Fronto-nasal suture	Anterior-most point	Middle-anterior-most point on the anterior contour	Middle point
Gnathion (Gn)	Contour of the bony chin	Anterior-inferior-most point	Middle-anterior-inferior-most point	Middle-inferior-most point

» Frankfurt plane: plane passing through the right and left porion points and the left orbitale (PoR, PoL - OrL).
» Midsagittal plane: plane referring to the junction of nasion and basion points, perpendicular to the Frankfurt plane. Used to evaluate gnathion deviation on transversal direction.

Three qualified professionals conducted the tomographic measurement of gnathion displacement in relation to the midsagittal plane at the diagnosis service center. Therefore, the error of the method was determined through intraclass correlation coefficient (ICC) to assess intraobserver and interobserver reliability. The three experienced professionals analyzed 10% of the tomographic scans at two different time intervals with a two-week interval between the first and second evaluations. Intraobserver ICC was 0.94 while interobserver ICC was 0.92 for the evaluated measurement, thus demonstrating reliability of the method. The average difference between observations was always smaller than 0.50 mm. The reliability on determining the presence of posterior teeth through panoramic reconstructions was also evaluated in these same tomographic scans by means of Kappa test. An index of 1.00 was obtained, thus indicating perfect agreement on the performed evaluations. 

SPSS version 20.0 software (IBM, Chicago, IL, USA) was used to analyze the collected data. Chi-square test (X^2^) was conducted with a 5% level of significance in order to evaluate the association between posterior tooth absence and mandibular asymmetries.

## RESULTS

**Figure 2:**
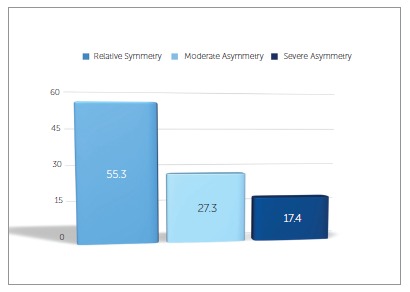
Prevalence of different degrees of mandibular asymmetries contemplated in this study.

Through analysis of the collected data, a few notes could be drawn. Regarding the degree of mandibular asymmetry, the occurrence of a relative symmetry, a moderate asymmetry, and a severe asymmetry was of 526 (55.3%), 260 (27.3%) and 166 (17.4%), respectively (Fig 2). 

Table 2 shows the characteristics of the sample as regards to sex, age, and gnathion displacement in relation to the frequency, as well as mean (ẋ), standard deviation (SD), and range (R) in each of the categories of mandibular asymmetry contemplated in the study. 

**Table 2:** Characteristics of the sample according to the degree of mandibular asymmetry.

	Degree of mandibular asymmetry			
	Relative symmetry (n = 526)	Moderate asymmetry (n = 260)	Severe asymmetry (n = 166)	Total sample (n = 952)
Sex, male n (%)	170 (32.3%)	90 (34.6%)	57 (34.3%)	317 (33.3%)
Age, years X ± SD; (R)	32.0 ± 11.7 (18-70)	31.7 ± 11.6 (18-75)	31.1 ± 9.2 (18-67)	31.5 ± 11.3 (18-75)
Gn displacement to MSP, mm X ± SD; (R)	0.76 ± 0.59 (0.00-2.00)	2.83 ± 0.57 (2.01-3.99)	7.05 ± 3.34 (4.01-21.49)	2.53 ± 2.68 (0.00-21.49)

Table 3 presents the chi-square test employed to assess the association between posterior tooth presence and degrees of mandibular asymmetry. It was observed that moderate and severe mandibular asymmetries occurred in a higher proportion in patients with unilateral posterior absence; however, there was no statistically significant difference between the analyzed groups (X^2^ = 2.245; *p* = 0.691 not significant).

**Table 3:** Association between posterior teeth presence and mandibular asymmetries.

	Degree of mandibular asymmetry n (%)			Total	*p* value
	Relative symmetry	Moderate asymmetry	Severe asymmetry		
Posterior teeth					0.691
All teeth	362 (55.1)	182 (27.7)	113 (17.2)	657 (69.0)	
Unilateral absence	64 (50.8)	37 (29.4)	25 (19.8)	126 (13.2)	
Bilateral absence	100 (59.2)	41 (24.3)	28 (16.5)	169 (17.8)	
Total	526 (55.3)	260 (27.3)	166 (17.4)	952 (100)	

## DISCUSSION

In this study, the prevalence of a relative symmetry, a moderate mandibular asymmetry, and a severe mandibular asymmetry was of 55.3%, 27.3%, and 17.4%, respectively. Moreover, it was observed that mandibular asymmetries do not present association with the absence of posterior teeth in adults. Thus, the hypothesis that loss of masticatory function caused by tooth absence could be associated with the occurrence of skeletal asymmetries was rejected. 

Previous studies have also evaluated the prevalence of mandibular asymmetry,^4^ ^,^ ^21^ ^,^ ^22^ but only a few of them have used a representative sample or have classified its different magnitudes. 

It is important to highlight that the sample comes from a service center for dental diagnosis and planning. Therefore, it does not depict adults in general, since this convenience sample was collected in retrospect based on patients that were referred for tomographic examination. Furthermore, both the cause and time of posterior tooth absence could not be determined, since it was determined by occlusion discontinuity assessed by panoramic reconstruction.

The approach adopted for this research evaluated skeletal symmetry by means of gnathion displacement in relation to the midsagittal plane because the literature reports that craniofacial asymmetry has menton deviation as one of its most remarkable characteristics. Additionally, diagnosis is more easily established through front-view analyses.^1^ ^,^ ^4^ ^,^ ^7^

Taking into account the intensity of menton deviation in relation to the midsagittal plane, it was possible to observe that slight mandibular symmetry was the most prevalent in the studied sample, followed by moderate asymmetry, leaving severe asymmetry as the least prevalent. 

Studies found in the literature present asymmetry prevalence between 12% and 37%;^4^ ^,^ ^5^ ^,^ ^21^ ^,^ ^23^ ^,^ ^24^ however, many of these studies assess mandibular asymmetry by means of visual methods or other radiographic methods. Moreover, most studies only classify asymmetry as present or absent; in contrast, the present study aimed at discriminating degrees of mandibular asymmetry, considering that different degrees usually demand different treatment approaches. 

The study conducted by Ramirez-Yañez et al,^5^ which evaluated mandibular symmetry in the panoramic radiographs of 327 children and classified them into four different categories, also found a smaller prevalence of severe asymmetries. Likewise, Masuoka et al,^3^ working with a sample of 100 asymmetric patients assessed by posterior-anterior cephalograms, noted a higher number of patients with relative symmetry, followed by patients with moderate asymmetry and lastly patients with severe asymmetry. 

Given that no face is perfectly symmetrical, it is accepted that mild facial asymmetry (also known as relative symmetry or insignificant asymmetry) may be considered normal, and often neither the patient nor the people around him are able to notice it. Moderate asymmetry, however, is commonly detected and may be treated in a compensatory manner, whether by means of adopting orthodontic or orthopedic approaches during adolescence. On the other hand, severe asymmetry concurrently compromises patient's function and esthetics. For this reason, it is usually corrected by combining orthodontic and surgical treatments as appropriate.^25^

Regarding the association between tooth absence and skeletal asymmetries, it is necessary, at this point, to confront our findings with studies that analyze the effects of mastication, occlusion, and tooth absence on lateral craniofacial development. The literature reports that an unbalanced occlusion and an asymmetric masticatory function may cause disharmonies between right and left sides of the mandible.^10^

In regards to the relation between masticatory efficiency and malocclusion, Omar et al,^12^ English et al,^26^ and Magalhães et al^27^ claim that occlusal problems negatively affect one's ability to crush and process food. Nevertheless, analyzing skulls of fetuses, infants, children, and adults, Rossi et al^28^ discovered that craniofacial asymmetry is statistically significant in fetuses and infants (prior to dentition); thus, the hypothesis that craniofacial asymmetry only appears after the establishment of the masticatory habit can no longer be maintained. 

As to the association between malocclusion and mandibular skeletal asymmetry, the results presented in the literature are fairly controversial. Kusayama et al^9^ reported that their sample exhibited a high correlation between occlusion anomalies and skeletal asymmetries. Similarly, Sezgin et al^29^ claim that Class II, Division 1 malocclusions seem to be more related to condylar asymmetries. However, Letzer and Kronman^30^ found no association between skeletal asymmetries and malocclusions. O'Byrn et al^31^ assessed mandibular asymmetry in adults with unilateral crossbite and did not find skeletal asymmetry in those patients. In a systematic literature review, Talapaneni and Nuvvula^32^ stated that an evidence-based conclusion could not be drawn about the association between posterior unilateral crossbite and structural mandibular asymmetry.

Studies specifically examining the effects of posterior tooth absence on skeletal asymmetry are very scarce in the literature. Caglaroglu et al^11^ analyzed 51 patients with early unilateral first molar extraction by means of posterior-anterior cephalograms. Subsequently, those subjects were compared to 30 patients with no missing teeth. The authors concluded that unilateral molar extraction during growth and development may result in both dental and skeletal asymmetries, especially in the lower third of the face. 

Halicioglu et al^10^ assessed 51 patients with early unilateral mandibular first molar extraction by means of panoramic radiographs. Patients were then compared to a control group consisting of 51 patients. The authors found that only the index of condyle asymmetry associated with the mandibular ramus presented differences between groups; however, the difference was so small that it was considered clinically insignificant. 

Halicioglu et al^13^ also studied the effect of bilateral premature loss of mandibular first molars by means of panoramic radiographs and did not report lateral asymmetries in those patients. 

It is thus observed that the basic processes determinant to the development of skeletal asymmetries remain unclear. Lack of controlled longitudinal studies prevents the establishment of a precise cause. Despite its cross-sectional design, the results of this study allow to determine the inexistence of an association between mandibular asymmetries and posterior tooth absence in adults, considering the methodology used and size of the sample. Hence, although some authors^12^ ^,^ ^26^ ^,^ ^27^ argue that the absence of posterior teeth may interfere negatively over dental occlusion and masticatory efficiency, it did not present statistical association with mandibular skeletal asymmetry in this study. Further studies should be conducted to better comprehend the several factors that could be related to skeletal asymmetries, as well as to attempt to determine the weight of genetics as an etiological factor of such alterations. 

## CONCLUSION

The analysis of the collected data led to the conclusion that the prevalence of a relative symmetry was of 55.3%, followed by moderate and severe asymmetries of 27.3% and 17.4%, in that order.

No association between mandibular asymmetries and the absence of teeth in the posterior region of the arch was observed. 
